# The Receptor for Advanced Glycation End Products (RAGE) Contributes to the Progression of Emphysema in Mice

**DOI:** 10.1371/journal.pone.0118979

**Published:** 2015-03-17

**Authors:** Nisha Sambamurthy, Adriana S. Leme, Tim D. Oury, Steven D. Shapiro

**Affiliations:** 1 Division of Pulmonary, Allergy, and Critical Care Medicine, Department of Medicine, University of Pittsburgh, Pittsburgh, Pennsylvania, United States of America; 2 Department of Pathology, Department of Medicine, University of Pittsburgh, Pittsburgh, Pennsylvania, United States of America; University of Miami, UNITED STATES

## Abstract

Several recent clinical studies have implied a role for the receptor for advanced glycation end products (RAGE) and its variants in chronic obstructive pulmonary disease (COPD). In this study we have defined a role for RAGE in the pathogenesis of emphysema in mice. RAGE deficient mice (RAGE-/-) exposed to chronic cigarette smoke were significantly protected from smoke induced emphysema as determined by airspace enlargement and had no significant reduction in lung tissue elastance when compared to their air exposed controls contrary to their wild type littermates. The progression of emphysema has been largely attributed to an increased inflammatory cell-mediated elastolysis. Acute cigarette smoke exposure in RAGE-/- mice revealed an impaired early recruitment of neutrophils, approximately a 6-fold decrease compared to wild type mice. Hence, impaired neutrophil recruitment with continued cigarette smoke exposure reduces elastolysis and consequent emphysema.

## Introduction

Chronic obstructive pulmonary disease (COPD) is a major cause of morbidity and premature mortality in the United States and an epidemic worldwide. Cigarette smoke exposure is a major risk factor in the development of COPD [1]. Emphysema is a major component of COPD that is characterized by the abnormal and irreversible enlargement of alveoli [2]. Progression of emphysema is attributed to increased inflammation, with elevated attendant oxidative stress, and protease activity, leading to cellular apoptosis [3] and loss of elastin fibers. Despite advances in understanding the cellular and molecular mechanisms mediating the development of the disease, the precise molecular pathways and mediators leading to emphysema is not definitively known.

Animal models of emphysema have been used to better understand the pathogenesis of COPD. Genetic engineering in mice has made it possible to manipulate gene expression to better understand its contribution to the pathobiology of the disease [4]. While mouse and human lung anatomy are comparable for the most part, certain differences do exist in that mice have sparse airway branching, fewer sub-mucosal glands in their trachea, lesser cilia lining their airways and fewer Clara cells [5]. These differences complicate translation of physiological effects from cigarette smoke exposure in the proximal airways of mice to humans, however alveolar enlargement resulting from chronic smoke exposure is readily apparent in many mouse strains and is likely to translate to human emphysema.

The receptor for advanced glycation end products (RAGE) acts as a pattern recognition receptor and belongs to the immunoglobulin superfamily [6]. The membrane bound form of RAGE (m-RAGE) is highly expressed in normal adult lung tissue and has been shown to localize to the basolateral membrane of differentiated alveolar type-I epithelial cells [7]. RAGE is also expressed on bronchial smooth muscle cells, vascular endothelial cells, alveolar macrophages and transitioning alveolar type-II epithelial cells in the alveolar parenchyma [8]. Several ligands are known to interact with RAGE, such as DNA binding high mobility group box 1 (HMGB1) [9], S100 protein family [10], advanced glycation end products (AGEs) [11], glycosaminoglycans [12], beta amyloid proteins [13] and extracellular matrix components like collagen I, collagen IV and laminin [14,15]. RAGE binding ligands like HMGB1 and S100A8/A9 complex also bind and signal through the toll-like receptor 4 (TLR4) [16–18]. The soluble form of the receptor (s-RAGE) exerts antagonistic effects by binding these ligands and preventing their signaling through membrane bound RAGE (m-RAGE) or other receptors like TLR4.

*In-vitro* studies, utilizing a human embryonic kidney cell line (HEK293) transfected with full length RAGE illustrated the role of RAGE in cell adhesion and spreading on collagen IV matrix [15]. These findings highlighted the contribution of RAGE to alveolar type-I epithelial (AT-I) cell adhesion and spreading, thereby facilitating gas exchange in the lung. The predominant expression of RAGE by AT-I epithelial cells [19] suggests its potential contribution to alveologenesis and the maintenance of normal lung homeostasis. Deregulation of RAGE expression on lung tissue has been observed in various animal models and clinical studies in diverse pulmonary disorders such as fibrosis [20,21], non-small cell lung adenocarcinoma [22,23], asthma [24], pneumonia [25] and acute lung injury [26,27]. Smokers with COPD have greater intensity of staining for RAGE in the alveolar walls of the lung [28]. Decreased levels of antagonistic soluble RAGE (s-RAGE) were detected in the bronchoalveolar lavage fluid (BALF) from the lungs of smokers with COPD [29]. RAGE expression was increased following *in vitro* exposure to cigarette smoke extract in rat (R3/1) cells and human (A549) cells [30]. Intratracheal delivery of cigarette smoke extract (CSE) induced RAGE expression in alveolar macrophages [31]. RAGE deficient macrophages exposed to CSE showed reduced active Ras and p38 MAPK and lesser nuclear translocation of pro-inflammatory nuclear factor κB (NF-κB) in comparison to their wild type counterparts[31]. Further RAGE deficient mice showed decreased activation of Ras in their lung tissue and reduced cytokine generation on chronic cigarette smoke exposure [32]. Transgenic mouse models that conditionally over-expressed RAGE in their alveolar epithelium displayed airspace enlargement, increased apoptosis, increased matrix metalloproteinase-9 (MMP-9) expression and decreased elastin expression [33]. Together these studies indicate a potential role for RAGE in the pathogenesis of COPD. However, the direct involvement and contribution of RAGE to the development of cigarette smoke induced emphysema has not been explored thus far. In the current study we exposed wild type and RAGE-/- mice to cigarette smoke to determine if RAGE was required for the development and progression of emphysema.

## Materials and Methods

### Ethics statement

The animal studies performed as part of this study conformed to the guide for care and use of laboratory animals of the national research council (NRC). The protocol used was reviewed and approved by the University of Pittsburgh Institutional Animal Care and Use Committee (IACUC) (Protocol number: 12101008). The animals were handled and euthanized with minimal discomfort and suffering. The mice were anesthetized using sodium pentobarbital and were sacrificed by carbon dioxide narcosis.

### Mice

RAGE-/- mice were originally generated from a founder RAGE gene targeted colony provided by Dr. A. Bierhaus (University of Hiedelberg, Germany) [34]. RAGE-/- mice were backcrossed 10 generations into the C57BL/6J background. Eight to twelve week old female mice were used in all the experiments designed. Age, sex and background matched wild type (C57BL/6J) mice were obtained from Jackson Laboratories.

### Cigarette smoke exposure

For long-term smoke exposure studies, RAGE-/- and wild type mice were subjected to the smoke of 4 unfiltered cigarettes per day (purchased from the University of Kentucky), 5 days a week over 6 months, using a smoking apparatus that delivers cigarette smoke to mice housed in individual chambers [35]. Mice tolerated cigarette smoke exposure without evidence of toxicity (carboxyhemoglobin levels ~ 10% and no weight loss). The controls in each group were exposed to room-air alone. These mice were caged separately and housed in the same facility as their smoke exposed counterparts. For acute smoke exposure experiments, RAGE-/- and wild type mice were exposed to smoke from 2 unfiltered cigarettes using the same smoking apparatus utilized in the chronic studies and later sacrificed at 4 hours post-cigarette smoke exposure.

### Respiratory mechanic measurements

We measured airway resistance, tissue damping, tissue elastance, dynamic resistance, dynamic elastance and dynamic compliance in response to methacholine challenge (Sigma, St. Louis, MO) using a computer-operated ventilator (Flexivent, Scireq, Montreal,QC). The mice were anesthetized with sodium pentobarbital (60mg/kg I.P.), a tracheotomy was performed and the animal attached to a mechanical ventilator. Each animal was given deep lung inflation to 30cmH_2_O distending pressure and baseline pressure-volume-flow measurements were captured. The animal was further exposed to PBS followed by increasing concentrations of methacholine (1, 3, 10 & 30mg/ml) using a nebulizer for 10 seconds through tracheostomy and the responses were recorded.

The responses recorded using a forced oscillation technique were then fitted to a constant phase model to compute the various parameters. We then plotted these parameters as a function of the log-transformed concentrations of methacholine. The slopes of the curves generated indicate differences in overall responses between strains and their treatments.

### Tissue processing for histology and morphometry

The mice were sacrificed by carbon dioxide inhalation, their chest wall exposed and their trachea was cannulated. The lungs were inflated with 10% normal buffered formalin (NBF) at a constant pressure of 25cmH_2_O pressure for ten to fifteen minutes via an intra-tracheal cannula, ligated and fixed for 24 hours before embedding in paraffin. Serial mid-sagittal sections were obtained for morphometric analysis.

### Morphometric analysis

Mid-sagittal sections were stained with a modified Gill’s stain and used to determine chord length (CL), an estimation of alveolar size, as previously described [35,36]. Ten randomly selected x200 fields per slide were photographed and the images analyzed using Scion Image software (Scion Corp., Frederick, MD) to estimate alveolar chord length. Airway and vascular structures were excluded from analysis.

### Western blotting to determine Caspase activation

Whole lung tissue isolated from the mice were first homogenized in isotonic buffer with CHAPS detergent (50 mM Tris-HCl, pH 7.4, 150 mM NaCl, 10 mM CHAPS) with protease inhibitors (100 μM 3,4-dichloroisocoumarin (DCI), 10 μM trans-epoxysuccinyl-L-leucylamido- (4-guanidino) butane (E-64), 2 mM o-phenanthroline monohydrate (all from Sigma) and then sonicated briefly. The protein concentration of the total lung homogenates was quantified using the BCA standard protein assay kit (Thermo Fisher). Lung homogenate samples (50μgs) were separated by SDS-PAGE and transferred to PVDF membranes as described previously [37]. The membranes were blocked overnight in 5% nonfat dry milk/PBST for an hour at room temperature. The membranes were then incubated with rabbit anti-mouse caspase-3 (1:1000; Cell Signaling) overnight at 4°C followed by anti-rabbit HRP IgG (1:2000) for 1 hr at room temperature. The membranes were washed with PBST (3x for 10 min) between primary and secondary antibody incubation. The reactive bands were visualized using the chemiluminescence method (SuperSignal West Pico, Pierce). The same membranes were reprobed with GAPDH (1:500; Cell Signaling) as loading controls. Densitometric analysis was performed using ImageJ to compare protein levels and the data analyzed by unpaired two-tailed student T test.

### Bronchoalveolar lavage fluid collection to determine leukocyte subsets

At 4 hours post- acute cigarette smoke exposure; batches of mice (n = 10–15mice/treatment/group) were sacrificed by CO_2_ narcosis. The chest cavity was exposed and the trachea of the mice was cannulated using a 22-guage intravenous catheter. The lungs were lavaged with 0.75 ml of PBS three times. The volume returned from each lavage was collected and recorded. The lavage fluid was centrifuged at 300 G for 5 minutes at 4°C and the supernatent collected. The cell pellet was then re-suspended in hypotonic solution to lyse red blood cells (RBC) and then centrifuged at 300 G for 5 minutes at 4°C. The supernatent containing lysed RBCs was discarded and the pellet resuspended in PBS. A small fraction of this cell supension was used to determine total cell counts using a hemacytometer and the remainder (approximately 200μl) was used to prepare the cytospin to determine the different subsets of leukocytes.

### Cytospin preparation and analysis

Cytospins prepared at 500rpm for 5 minutes, allowed to air dry and then stained with a modified Romanovsky stain. The slides were rinsed, allowed to air-dry and imaged. Five to ten random bright-field images were quantified per mouse (approximately 300 cells) depending on the total number of cells recovered. Cell populations were quantified and expressed as percentages, which were used to estimate the numbers of cell of a specific type in the total collected population.

### Statistical analysis

The data was quantified and analyzed using Graphpad Prism 5 (Graphpad Prism Inc., La Jolla, CA) and Minitab 17 (Minitab Inc., State College, PA). The results were represented as means ± standard error mean. Kruskal Wallis test was used to analyze chord length values (non-normally distributed) of mice exposed to cigarette smoke and room air. This test was used to examine the null hypothesis that all populations have identical distribution functions. A p-value≤0.05 indicated significant difference between the populations compared. These results have been tabulated in Table 1. In the case of normally distributed data, an unpaired 2-tail student T-test was performed to determine statistical significance between strains and treatments. A p-value≤0.05 was considered statistically significant and indicated by an asterisk (*) in graphical representation.

**Table 1:** Quantification of airspace enlargement using lung morphometry.

Batch	Group	N/group	CL (SEM)	% Increase with Sm	P Value (vs. NSm)	P Value (vs. WT-NSm)
1	**WT (NSm)**	5	26.19(0.16)	-	-	-
	**WT (Sm)**	5	29.72(0.38)	13.47	0.009	-
	**RAGE-/- (NSm)**	5	30.13(0.12)	-	-	0.009
	**RAGE-/- (Sm)**	4	31.02(0.60)	2.95	0.327	0.014
2	**WT (NSm)**	5	28.38(0.52)	-	-	-
	**WT (Sm)**	5	32.36(0.99)	14.02	0.009	-
	**RAGE-/- (NSm)**	5	32.22(0.45)	-	-	0.009
	**RAGE-/- (Sm)**	4	34.82(0.52)	8.06	0.006	0.006

Mean chord length (CL) data from 2 independent experiments of chronic cigarette smoke exposures. Wild-type (WT) mice were C57BL/6J background, congenic with the RAGE-/- mouse strain. The CL, the standard error of the mean (within the parentheses) as well as the percentage increase in CL with smoke exposure is displayed. The Kruskal-Wallis test was used to derive the p-value indicated. A p-value≤0.05 indicated that the groups compared have significantly different distribution functions.

## Results

### Absence of RAGE causes enlarged alveolar dimensions at baseline, but protection from progressive enlargement on chronic cigarette smoke exposure

**Fig 1:**
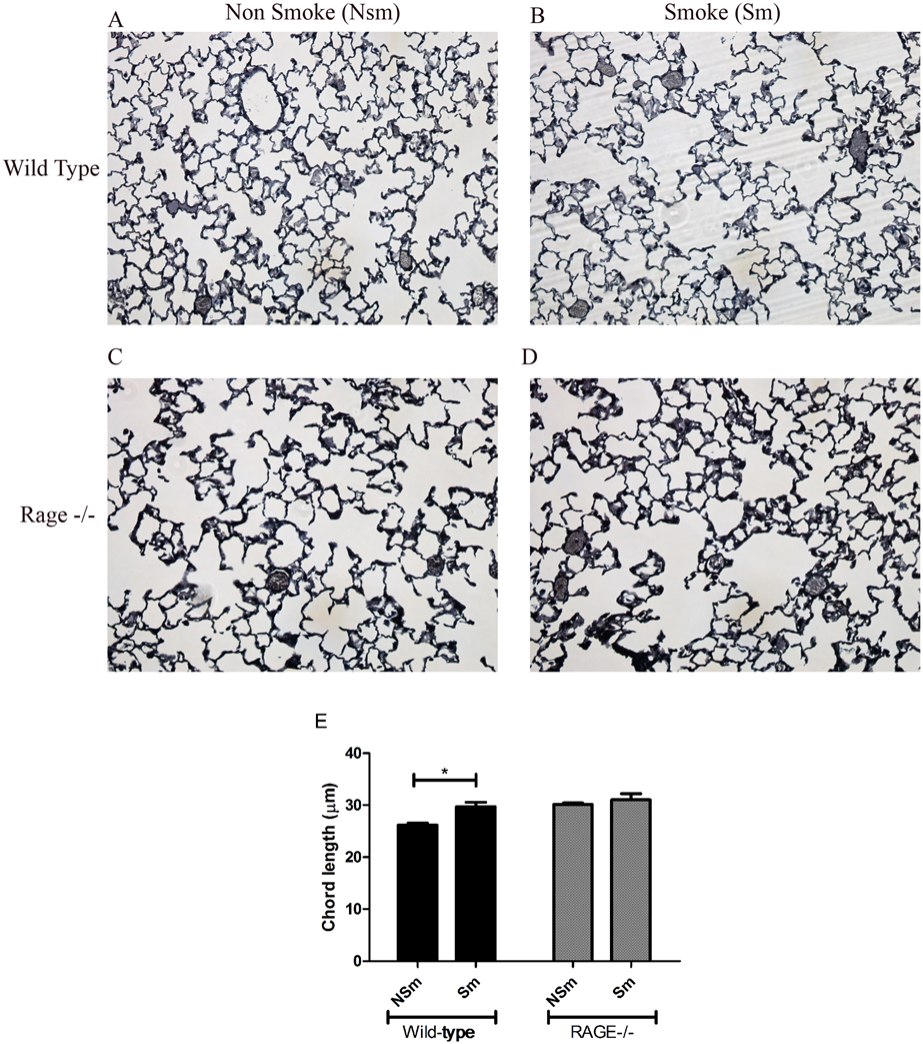
Lack of RAGE partially protects from progressive airspace enlargement. RAGE-/- and wild type mice were exposed either to cigarette smoke or room-air and sacrificed after 6 months. **Panel A-D** show representative images of gills stained sections of the lungs from the different treatment groups. **Panel E** is a graphical representation of the mean chord lengths (μm) from the first experimental group. * Indicates a p-value < 0.05 by Kruskal-Wallis test. The bars indicate the standard error mean (SEM). Note, RAGE-/- mice have larger chord lengths at baseline but are protected from cigarette smoke-induced enlargement compared to their wild type counterparts.

Age and sex matched RAGE-/- and wild type (C57BL/6J background) mice (n = 4–5 per treatment group) were exposed to either cigarette smoke or room-air for 6 months. Morphometric analysis was performed to examine alveolar dimensions (detailed in methods). Histological examination of cigarette smoke exposed wild type mice (Fig. 1B) indicated alveolar enlargement when compared to their room air exposed controls (Fig. 1A). Morphometric analysis confirmed that wild-type mice exposed to cigarette smoke showed a 13.5% increase (p-value = 0.009) in CL (29.7±11.3μm) as compared to their air-exposed controls (26.2±4.2μm). These results were replicated in a second batch of mice (n = 5–6 mice per treatment group), where a 14% increase in CL was observed in smoke exposed wild type mice compared to their controls (Table 1).

Of note, RAGE-/- mice had enlarged airspaces at baseline in the absence of smoke exposure (Fig. 1C) compared to wild type (Fig. 1A). The mean CL of RAGE-/- mice exposed to room air (30.1 ±3.6 μm) was significantly higher (p-value = 0.009) than room air-exposed wild type mice (26.2±4.2 μm) (Table 1).

Cigarette-smoke exposure in RAGE-/- mice (31.2±18.6μm) did not lead to a significant increase in CL as compared to their air-exposed controls (30.1±3.6μm) (Fig. 1 and Table 1). Together these results imply that the absence of RAGE contributes to the development of emphysema. Further RAGE-/- mice showed significant protection from cigarette smoke induced airspace enlargement when compared to their wild type counterparts. Hence, airspaces in RAGE-/- mice are enlarged at baseline; but protected from further enlargement in response to cigarette smoke.

### Lung tissue elastance was maintained in RAGE-/- mice exposed to cigarette smoke

**Fig 2:**
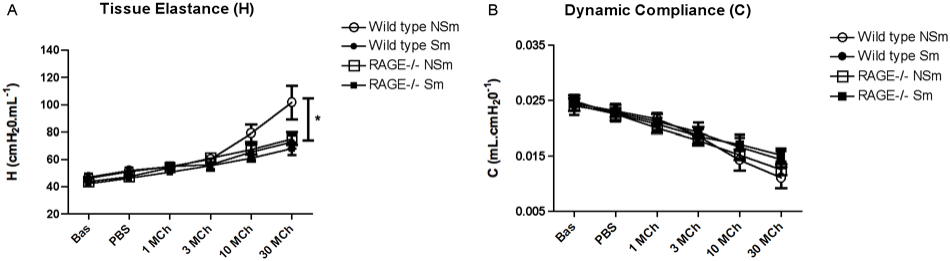
Cigarette smoke exposed RAGE deficient mice show no reduction in lung tissue elastance on bronchocontriction when compared to their air-exposed controls. Respiratory mechanics of RAGE-/- mice and wild type mice (n = 5–6 mice per strain per treatment group) previously exposed to cigarette smoke or room air was assessed using a mechanical ventilator (Flexivent) as described in the methods section. **Panel A** indicates a significant reduction in lung tissue elastance in wild type smokers (●) as compared to the air-exposed controls (◯) on bronchocontriction. However, RAGE-/- mice show no reduction in lung tissue elastance (H) on cigarette smoke exposure (■) (or methacholine challenge) compared to their controls (□). **Panel B** shows dynamic lung compliance (C) was not significantly altered by methacholine challenge in either mouse strain irrespective of cigarette smoke exposure. Standard 2-tailed student t-tests were performed and p-value < 0.05 indicate statistical significance (as denoted by *).

Pulmonary function testing was performed on RAGE-/- (n = 5–6 mice/treatment group) and wild type mice (n = 5 mice/treatment group) with and without exposure to chronic cigarette smoke. Wild type smoke exposed mice showed a significant dose-dependent decrease (p-value = 0.048) in lung tissue elastance (H) in response to methacholine challenge when compared to their room-air exposed controls (Fig. 2A). Although emphysema was too mild to detect differences in tissue elastance (H) at baseline, addition of methacholine brought out significantly reduced elasticity with smoke exposure, which correlated with the significantly enlarged alveoli observed in these mice on morphometric analysis (Fig. 1E & Table 1).

RAGE-/- mice at baseline did not display any difference in tissue elastance (H) irrespective of their exposure to cigarette smoke or room-air when compared to their wild-type counterparts. However, in response to challenge with 30mg/ml of methacholine the RAGE-/- air-exposed mice (Fig. 2A) displayed a trend towards decreased tissue elastance (75.29 cmH_2_O/ml) as compared to the air-exposed wild-type mice (101.65 cmH_2_O/ml). Chronic smoke exposure in RAGE deficient mice led to no change in H compared to their room-air exposed controls. RAGE-/- mice irrespective of their exposure to cigarette smoke or room-air had similar changes in H when compared with wild-type chronic smoke exposed mice on bronchodialation. While differences in H were observed with smoke exposure, we did not detect any changes in dynamic compliance, C (Fig. 2B).

### Cigarette smoke-induced apoptosis is RAGE-dependent

**Fig 3:**
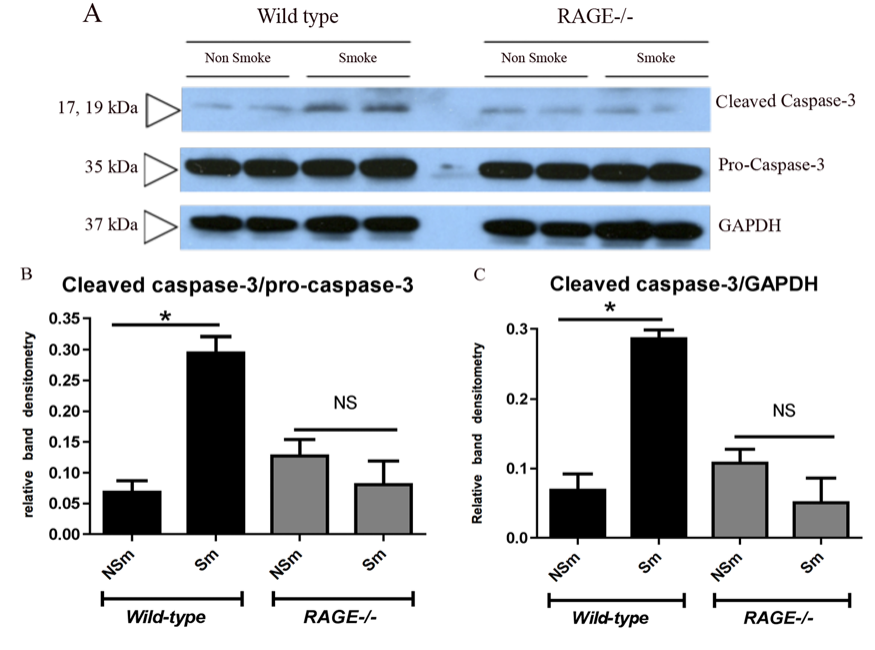
RAGE expression contributes to apoptosis with long-term smoke exposure. Lung homogenates (50μg per well) harvested from RAGE-/- and wild type mice (n = 2/strain/treatmentgroup) exposed either to chronic cigarette smoke (Sm) or room air (NSm) was separated by SDS-PAGE and immunoblotted for caspase-3. Glyceraldehyde 3-phosphate dehydrogenase (GAPDH) with a molecular size of 37kDa was used as an internal control. Panel A- Western blot showing cleaved Caspase-3 and Pro-Caspase-3 bands in total lung homogenates. Cigarette smoke exposure led to significantly elevated caspase-3 cleavage in wild type but not RAGE-/- mice, when normalized either to Pro-Caspase-3 levels (Panel B) or GAPDH levels (Panel C). Band densities were analyzed using ImageJ and unpaired 2-tailed student T-test was used to analyze the normalized band intensities (*p<0.05).

Alveolar endothelial and epithelial apoptosis is well characterized in cigarette smoke-induced COPD that correlates with the loss of alveolar tissue [38]. To investigate the role of RAGE in mediating cellular damage in response to cigarette smoke exposure, total lung homogenates from RAGE-/- (n = 2 per treatment group) and wild-type (n = 2 per treatment group) mice were probed for caspase-3 (a marker of apoptosis) by western blotting. Pro-caspase3 (35kDa) levels were similar between the wild type and RAGE-/- mice irrespective of their exposure to cigarette smoke or room air (Fig. 3A). However wild-type mice exposed to cigarette smoke had significantly elevated cleaved caspase-3 levels when compared to their air-exposed controls (Fig. 3B and C). In the absence of RAGE, no alteration in cleaved caspase-3 levels was observed in total lung homogenates from cigarette smoke-exposed mice in comparison to their controls. Band densitometry analysis of cleaved Caspase-3 normalized to either Pro-caspase-3 (Fig. 3B) or GAPDH (Fig. 3C) levels substantiate these findings. Subsequent repetition of this blot confirmed these observations in a total of n = 6 mice per strain per treatment group.

### Impaired neutrophil recruitment is observed in RAGE-/- mice in response to acute cigarette smoke exposure

Emphysema results from inflammatory cell-mediated elastolysis. Moreover RAGE has been shown to mediate the adhesion and recruitment of inflammatory cells [39]. Upon exposure to cigarette smoke, neutrophils are recruited within hours and return to normal within 24 hours. Macrophages and lymphocytes are not increased after acute smoke exposure, but appear to accumulate over time.

We did not detect any difference in macrophage recruitment or MMP-12 production (unpublished data) with chronic smoke exposure in the RAGE-/- mice compared to their air-exposed controls.

**Fig 4:**
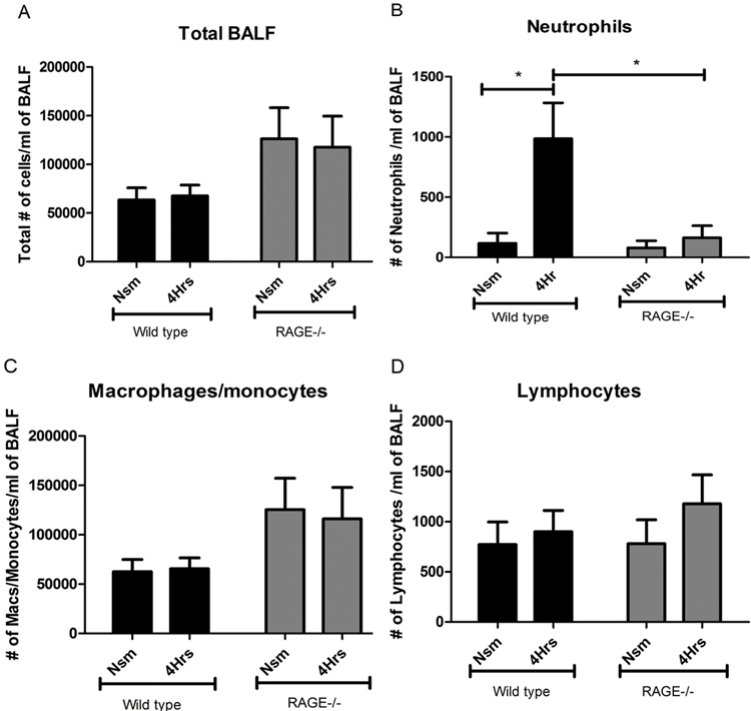
RAGE mediates early recruitment of neutrophils in response to acute cigarette smoke exposure. The bronchoalveolar lavage fluid (BALF) collected from the lungs of RAGE-/- (grey bars) and wild type (black bars) mice (n = 8–15 per strain per group) were used to stain and quantify cells that had migrated into the fluid lining the alveolus. **Panel A** indicates the total cell counts obtained from the BALF of the different treatment groups on acute cigarette smoke exposure. RAGE-/- mice displayed significantly reduced numbers of neutrophils at 4 hours post- smoke exposure as compared to their wild type counterparts, as shown in **Panel B**. There was no significant alteration observed in the number of macrophages or monocytes (**Panel C**) and lymphocytes (**Panel D**) recruited in either the RAGE-/- or wild type mice at 4 hours post-acute smoke exposure. The error bars indicate the standard error mean (SEM) within each group/treatment/time point (* p≤0.05 indicates statistical significance by unpaired 2-tailed student t-test).

Acute smoke exposure did not alter total BALF cell counts (Fig. 4A), however there was significant neutrophil accumulation in wild type mice, but not in RAGE-/- mice (Fig. 4B) (Note, neutrophils account for ~1% of BAL cells, hence not reflected in total counts). Specifically, at four hours post-acute smoke exposure wild type mice (n = 10) showed an eight-fold increase (p = 0.018) in neutrophils recruited when compared to their room-air exposed (n = 10) controls (Fig. 4B). Macrophage, monocyte and lymphocyte counts were unaltered at 4 hours post-acute smoke exposure in the wild-type mice as compared to their room-air exposed controls (Fig. 4C and Fig. 4D).

In contrast, RAGE-/- mice showed no significant increase (p = 0.475) in neutrophils at 4 hours post-smoke exposure (n = 15) (Fig. 4B) compared to their room-air exposed (n = 15) controls. There was no significant difference in the macrophage or monocyte and lymphocyte counts from the BALF of RAGE-/- mice at 4 hours post-acute smoke exposure when compared to their air-exposed controls (Fig. 4C and Fig. 4D). Hence, RAGE is required for cigarette smoke-induced neutrophil recruitment.

## Discussion and Conclusions

Upon long-term exposure to cigarette smoke, despite having enlarged airspaces at baseline, RAGE-/- mice did not develop significant airspace enlargement compared to wild type mice. Protection from smoke-induced emphysema in RAGE-/- mice was based upon morphometric analysis with less increase in airspace dimensions, respiratory mechanics with less loss of lung recoil (or less reduction in tissue elastance), and less cellular damage and apoptosis. The results further suggest that the partial protection from emphysematous changes observed was due to impaired early neutrophil recruitment in the absence of RAGE expression.

Enlarged alveolar dimensions in RAGE-/- mice not exposed to cigarette smoke suggest an involvement of RAGE during alveolar development. RAGE is expressed highly on alveolar type-I (AT-I) cells under normal conditions [19,40] serving as a marker of terminal differentiation on these cells [15]; hence the absence of RAGE may contribute to defective differentiation of the alveolar epithelial cells lining the lung parenchyma. In fact, transgenic mice that over-express RAGE in SP-C expressing alveolar type—II epithelial cells displayed significant alveolar hypoplasia, alterations in alveolar differentiation and weakened basement membrane [41,42]. In addition mouse models that over-express RAGE have poor alveolar septation during post-natal development [33,43]. These findings combined with our observation of enlarged airspaces in the RAGE-/- mice (Fig. 2C) point toward a requirement for RAGE in normal alveolar development and differentiation. Of note, airspace enlargement due to developmental abnormalities such as impaired alveolarization must be distinguished from destruction of mature airspaces due to inflammation and destruction. In fact, with larger baseline alveoli, this would tend to increase alveolar wall tension predisposing to greater percent increase in alveolar dimensions with cigarette smoke exposure as opposed to the protection from further airspace enlargement that we observed in RAGE-/- mice.

RAGE has been implicated as having a role in COPD. Clinical studies demonstrated increased staining for advanced glycation end products (AGE) and RAGE in lung tissue sections from COPD patients with an FEV1 <80% predicted, equivalent to GOLD stages 2–4 [28]. Additionally, this increased intensity of RAGE staining was significantly elevated in the alveolar walls when compared to the airways. Further, lower plasma levels of soluble RAGE (sRAGE) correlated with increased severity of emphysema in COPD patients when compared to controls who lacked airflow obstruction [29]. These findings were corroborated by observations in a larger COPD patient cohort [44]. *In vitro* studies performed in a rat alveolar type-I cell line (R3/1), human alveolar type-II cell line (A549) and a murine macrophage-like cell line (RAW246.7) showed up-regulated expression of RAGE and RAGE ligands upon exposure to cigarette smoke extract [30]. In this study we directly confirmed a role for RAGE in the pathogenic progression of emphysema by using RAGE deficient mice exposed to chronic cigarette smoke and show that they are partially protected from smoke induced airspace enlargement.

Inflammation associated destruction of lung tissue, particularly elastin, has been considered the basis for the pathogenesis of emphysema [45]. The elastase: antielastase hypothesis has stood the test of time for over 50 years [46]. With respect to inflammation, acute cigarette smoke exposure leads to transient recruitment of the short-lived neutrophils to the lung that peaks at 4 hours followed by loss within 24 hours. Hence, despite representing a small percentage of the total cells recovered by BAL, there is a large contribution of neutrophil burden elicited by smoking. Upon prolonged smoke exposure, longer-lived lymphocytes and macrophages accumulate in the lung [47].

Both neutrophils and macrophages interact to promote emphysema, largely through neutrophil elastase (NE), macrophage elastase (MMP-12) and perhaps MMP-9 expressed in both neutrophils and macrophages [36]. In our study, RAGE deficiency impairs neutrophil accumulation but not macrophage numbers and MMP-12 content (not shown). RAGE appears to be essential for early neutrophil recruitment as demonstrated by the fact that RAGE-/- mice have only ~17% the neutrophils in response to cigarette smoke exposure in contrast with wild-type mice. Hence, these studies further support the importance of neutrophils in emphysema and suggest that RAGE is involved in cigarette smoke induced early recruitment of neutrophils into the lung.

RAGE when engaged by its ligands has been shown to activate intracellular NF-κB mediated chemokine/cytokine transcription, thereby perpetuating inflammatory cell recruitment in other acute and chronic inflammatory disease models [48,49]. In addition AGE-RAGE signaling may up-regulate the expression of several adhesion molecules through NF-κB activation namely E-selectin, intercellular adhesion molecule-1 and vascular adhesion molecule-1 specifically on endothelial cells [50,51]. In fact, RAGE has been identified as a counter-receptor for the leukocyte β2 Integrin Mac-1 facilitating its adhesion and recruitment across the vasculature [39].

To examine the importance of RAGE to cigarette smoke related neutrophil recruitment, we analyzed pro and anti-inflammatory cytokines such as tumor necrosis factor alpha (TNFα), Interferon gamma (IFNγ), IL-6 and IL-10 as well as chemokines including MIP-2, KC and MCP-1 in lung tissue homogenates and BALF. On analysis, we observed no significant differences in their levels of expression while comparing RAGE deficient mice to their wild-type counterparts (not shown). Either the changes were too subtle to detect, or alternative mechanisms exist in this model that require further investigation.

The contribution of extracellular matrix destruction versus cellular apoptosis has been debated. Clearly loss of alveolar tissue and consequently enlarged airspaces requires both the loss of structural cells and extracellular matrix (ECM). Whether the events are separate or one leads to the other (and which one initiates) is less clear. However it is plausible that the loss of ECM leads to anoikis (death following matrix detachment). Alternatively, cell death could lead to inflammation. In this study, we observed significantly increased apoptosis (increased cleaved caspase-3) in the wild type mice exposed to cigarette smoke for 6 months that correlated with emphysema. However, the RAGE-/- mice did not show any alteration in cleaved caspase-3 (apoptosis) on chronic cigarette smoke exposure when compared to their room air exposed controls (Fig. 3B and C). This reduced cellular damage observed in the RAGE-/- mice may attribute to the partial protection observed on morphometric analysis and physiologic assessment indicating no reduction in lung tissue elastance. Furthermore, this is consistent with previous studies that have found a role for RAGE in promoting apoptosis in other lung models [52].

The current study adds RAGE to a growing list of mediators of emphysema and confirms the importance of neutrophil recruitment. Further understanding of neutrophil recruitment mechanisms could lead to novel therapy that is badly needed for this devastating and common disease. As the era of precision medicine arrives, RAGE polymorphisms could also be a factor that identifies smokers who are either resistant or susceptible to COPD [53]. Single nucleotide polymorphisms in the minor allele (T allele) within exon 3 of RAGE gene, which converts a glycine at the 82^nd^ position into serine (G82S) present within the ligand-binding pocket increases affinity for AGE ligands and enhances its ligand binding function [54,55]. A recent clinical study in a Chinese population suggested that smokers with the G82S polymorphism in their RAGE gene had an elevated risk of developing COPD[56]. This finding along with future studies holds the promise of better evaluation of individual susceptibilities to this disease and its possible prevention.
